# Relationship between Respiratory Muscle Function and Postural Stability in Male Soccer Players: A Case-Control Study

**DOI:** 10.3390/healthcare9060644

**Published:** 2021-05-29

**Authors:** Felipe León-Morillas, Carlos Lozano-Quijada, Miguel Ángel Lérida-Ortega, Martha Cecilia León-Garzón, Alfonso Javier Ibáñez-Vera, Silvana Loana de Oliveira-Sousa

**Affiliations:** 1 Department of Physical Therapy, Catholic University of Murcia UCAM, Avenida de los Jerónimos, 30107 Murcia, Spain; fleon@ucam.edu (F.L.-M.); mcleon@ucam.edu (M.C.L.-G.); 2 INTRAFIS Research Group, Department of Pathology and Surgery, Center of Translational Research in Physi-otherapy, University Miguel Hernandez of Elche, Campus of Sant Joan d’Alacant, 032550 Elche, Spain; 3 Department of Health Sciences, Campus las Lagunillas, University of Jaen, 23071 Jaen, Spain; malerida@ujaen.es (M.Á.L.-O.); ajibanez@ujaen.es (A.J.I.-V.); 4 Hospital San Agustín, Andalusian Health Service, Avenida de San Cristóbal, 23700 Linares, Spain; 5 Escuela de Osteopatía de Madrid, Calle Saturnino Calleja, 28002 Madrid, Spain; 6 Department of Physical Therapy, Universidad de Murcia, Avenida Teniente Flomesta, 30003 Murcia, Spain; soliveira@um.es

**Keywords:** inspiratory muscle strength, endurance respiratory, postural stability, center of pressure, soccer players

## Abstract

The important role of postural stability in exercise performance has been determined by several authors. Despite this, few studies have analyzed the relationship between respiratory muscles’ strength and postural stability in athletes. For this reason, the aim of this study was to investigate the relationship between postural stability and respiratory muscles’ function in male soccer players. A case-control study was conducted over twenty-eight healthy men (18 soccer players; 10 non-athletes). Inspiratory muscle strength (MIP) and respiratory resistance (MVV) were obtained through a digital spirometer. Stability variables were obtained in standing position on a stabilometric platform and in open and closed eyes conditions. The area and length of the center of pressures and displacements in the X and Y range were analyzed. Pearson’s coefficient was used to measure the linear correlation between MIP, MVV and stabilometric variables. In the soccer players’ group, MIP and MIP % predictive were inversely correlated with length (*r* = −0.535 and *r* = −0.585; *p* < 0.05) and X range (*r* = −0.527 and r = −0.560; *p* < 0.05), whereas MVV was directly correlated with length (*r* = 0.606; *p* < 0.01) and Y range (*r* = 0.558; *p* < 0.05). Our results show that the greater the inspiratory muscle strength, the less displacement of the pressure center, while at higher respiratory rates there is greater displacement.

## 1. Introduction

Postural stability is an essential motor skill, based mainly on muscle synergies, which act to minimize the displacement of the center of pressure (CoP) and maintain an upright posture, proper orientation and adequate locomotion [1]. In a sport like soccer, postural talent might be considered as an indicator of performance [2]. During a soccer match, players must use their motor skills and control their posture, while also gathering visual information about other team members as well as opponents. Motor skills on show include frequently performed lower extremity passing, shooting and dribbling skills with soccer cleats on a grass surface [3]. Due to the conditions of the game itself, for soccer players, it is essential to have a good balance to control, pass or shoot the ball. Furthermore, players must conserve balance as opposing players try to take the ball away from them [4].

Postural stability can be measured by assessing an individual’s postural sway through changes in the center of pressure. Various external and endogenous factors have been identified as a source of postural disturbance, such as respiratory movements [5,6,7], tendon vibration [8], heartbeats [9], lower limb compression [10] or cooling [11]. The biomechanical changes of the torso during breathing movements, interfere on postural way, reducing by apnea [12] and increasing by hyperventilation [13]. Furthermore, the pattern of recruitment of inspiratory muscles also influences respiratory-related disturbance. Hamaoui et al. [5] showed that “rib cage breathing” (which favors the intercostal and neck muscles over the diaphragm) creates more postural disturbances than “abdominal breathing” (which favors the diaphragm).

Several authors have demonstrated that diaphragm, in addition to maintain the ventilation, helps to support the stability of the trunk: in coordination, the diaphragm and the abdominal muscles produce a hydraulic effect in the abdominal cavity which assists spinal stabilization by stiffening the lumbar spine through increased intra-abdominal pressure [14,15,16,17]. However, when there is weakness and/or an increase in respiratory demand (an inspiratory loading task) the role of the diaphragm in low back stability declines, especially due to early muscle fatigue [18]. For this reason, during intense exercise such as in a soccer game, respiratory muscle fatigue generated by increased demand can dramatically affect the postural function of the diaphragm.

Surprisingly, despite the copious literature on postural stability in athletes, few studies have investigated the role of respiratory muscle strength. Some studies conducted in patients with respiratory or neurology diseases [16,19,20] have shown that inspiratory muscle weakness can be correlated with greater oscillations in CoP. Therefore, the objective of this study is to analyze the relationship between postural stability and respiratory muscles function (strength and endurance) in soccer players.

## 2. Materials and Methods

### 2.1. Study Design

The present investigation was a case-control study conducted to examine the relationship between respiratory muscle function (strength and endurance) and postural stability in soccer players. The study was approved by the Catholic University of Murcia Research Ethics Committee. The STROBE statement for observational studies was adopted [21]. The data collection and the development of the study took place during the months of February and May of 2018.

### 2.2. Participants

Thirty-five healthy male subjects were evaluated as potential participants in the study: a group of 22 soccer players (G1), members of the UCAM under−23 university team who followed an identical training regimen; and a group of 13 non-athletes (G2), who exercised with below-moderate activity (e.g., walking, minimal or no activity), less than 2 sessions per week, each week. Eligibility criteria were healthy males between 18–25 years old, without respiratory and/or cardiac pathologies and who had not previously performed respiratory training. A physical assessment and interview were initially conducted for all subjects to collect anthropometric and demographic data. All the participants signed an informed consent form.

### 2.3. Measurement Variables and Procedures

#### 2.3.1. Respiratory Muscle Function

A digital spirometer (Datospir Touch Easy Spirometer, Sibelmed, Spain) was used to measure the inspiratory muscle strength and respiratory endurance. The inspiratory muscle strength was evaluated indirectly through the measurement of the maximum inspiratory pressure in the mouth (MIP cm H_2_0, MIP% predictive). With the individuals in a sitting position and wearing a nose clip, they were asked to perform a maximal expiration (near residual volume), followed by a maximal inspiration (near total lung capacity). The highest value obtained in the three trials that did not differ by more than 10% from the others was selected to establish MIP [22]. Respiratory muscle endurance was measured through maximal voluntary ventilation (MVV liters, MVV bpm). Subjects in the same position, were asked to breathe as fast and deep as possible for 15 s. Three maneuvers were carried out with 1-min rest between them, and the best value among the three maneuvers was chosen. Measurements of MIP and MVV were carried out 1 day apart to avoid fatigue of the respiratory muscles. To reduce errors in the evaluation of these variables, all measurements were made by the same researcher, an expert in respiratory physiotherapy.

#### 2.3.2. Stabilometric Variables

To measure bipedal postural stability, the outcome measures of this study were different parameters of the CoP displacement: area covered by the CoP (area), length of displacement of CoP (length), standard deviation of mediolateral position (X range) and anteroposterior position (Y range). All measurements were conducted with a measuring frequency of 100 Hz. A Freemed baropodometric platform (Rome, Italy) and Free-Step v.1.0.3 software (Rome, Italy) were used to measure stabilometric parameters with a good level of reliability [23]. The platform’s surface is 555 × 420 mm, with an active surface of 400 × 400 mm and 8 mm thickness.

**Figure 1:**
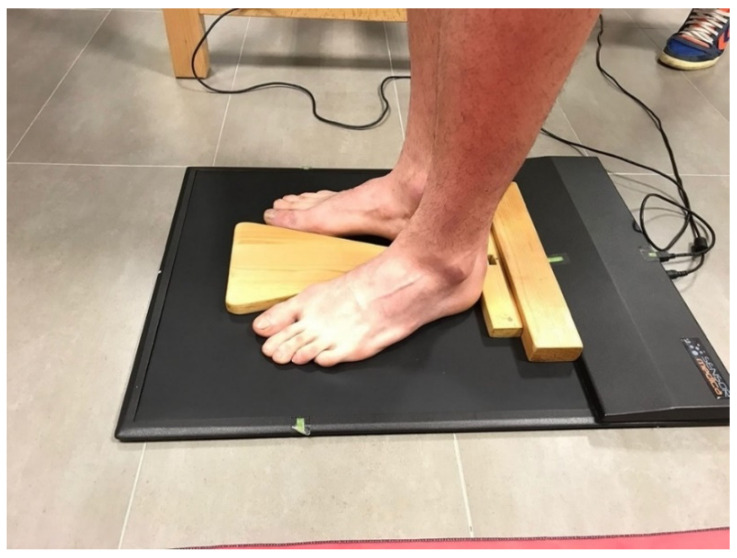
Wooden spacer for position standarization.

The participants had to stand barefoot on the baropodometric platform and with their feet at a 15-degree angle from the sagittal plane and heels separated by 2 cm. To ensure the initial standardization of this position, we have designed and used a wooden spacer, which was removed once the position was achieved (Figure 1). Measurements were made first in the open eyes condition (OE) and then closed eyes (OC) for 90 s to evaluate postural control with and without visual input. The measurement conditions were reproduced exactly for each group (soccer players and control group).

All the evaluations were carried out in the morning, in a research room at the UCAM, with an ambient temperature of 25 °C. The investigators responsible for the evaluations were experienced and previously trained.

### 2.4. Statistical Analysis

The IBM SPSS statistics 24^®^ program was used to record and analyze the data. The Kolmogorov-Smirnov test was used to evaluate the normality of the distribution of variables. To compare the homogeneity of the groups we used the t-student test. Pearson’s correlation coefficient was used to analyze the relationship between the respiratory muscle function (MIP and MVV) and stabilometric variables (area, length, X range and Y range). Statistical significance was set with a value of *p* < 0.05.

## 3. Results

**Figure 2:**
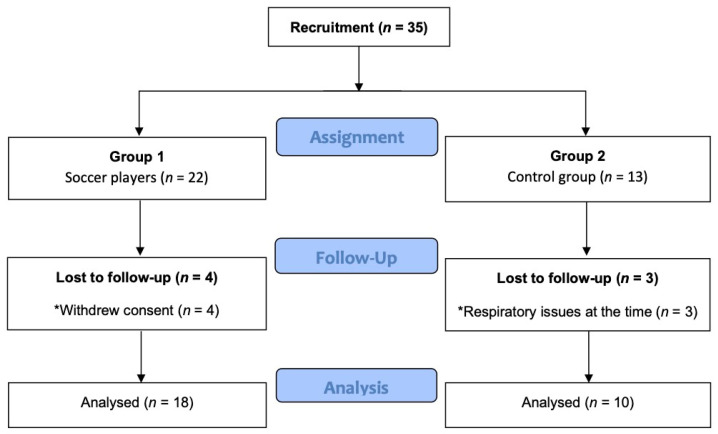
Flow diagram for recruitment process of the participants.

Of the 35 subjects initially recruited (22 soccer players, 13 non-athletes), 4 players finally decided not to participate, and 3 non-athletes had respiratory issues (suffered from common cold) at the time of the evaluation. Figure 2 describes the recruitment process of the participants.

Table 1 shows the general descriptive characteristics of the participants. There were no significant differences between the groups for most of the variables, except for maximum voluntary ventilation (bpm). The soccer players obtained a mean ± SD of 99.37 ± 33.45 and the control group of 175.74 ± 26.38 (*p* < 0.001).

The correlation coefficients between respiratory muscle function and stabilometric variables in soccer players and the control group is illustrated in Table 2. For the soccer player group, there was an inverse correlation between respiratory muscle strength and stabilometric variables in the closed eyes condition. The MIP and MIP % predictive were correlated with length (*r* = −0.535 and *r* = −0.585; *p* < 0.05; respectively) and lateral range (*r =* −0.527 and *r =* −0.560; *p* < 0.05; respectively). On the other hand, there was a direct correlation between respiratory muscle endurance and stabilometric variables in the open eyes condition. The MVV (bpm) was correlated with length (*r* = 0.606; *p* < 0.01) and anterior-posterior range (*r =* 0.558; *p* < 0.05). No significant correlations were observed between the variables studied for the control group.

## 4. Discussion

The objective of this study was to investigate whether respiratory muscle function was associated with postural stability in soccer players. We found that greater values of inspiratory muscle strength are associated with shorter path length and less lateral displacement in the closed eyes condition. On the other hand, we also demonstrate that the higher the respiratory rate in the MVV test, the greater the area and the greater anteroposterior displacement.

Using a platform of pressures, stabilometry assesses body sway through quantification of CoP anteroposterior and lateral movements during the standing position. Generally the measurement of CoP motion with a platform of pressures are considered the gold standard of balance [24]. Many investigators have attempted to understand which factors affect postural stability in athletes because this parameter correlates strongly with injuries [25]; however, the role of respiratory muscle strength in postural stability has not been explored.

In the present study, we demonstrate that inspiratory muscle strength is inversely related to standing postural stability in soccer players. These findings are consistent with previous research conducted in clinical settings. Penafortes et al., demonstrated that in cystic fibrosis the MIP was inversely correlated with the lateral standard deviation (*ρ* = −0.61; *p* < 0.05) [20]. Kocjan et al. [26], using other functional parameters of the diaphragm, closely related to its strength, found that greater values of diaphragm inspiratory thickness, diaphragm thickness fraction and diaphragm movement were associated with shorter path length and smaller ellipse field. In stroke patients, Lee et al. [19], found improvements in the stability limits of the CoP after training of the respiratory muscles.

The diaphragm, the main inspiratory muscle, participates in postural regulation through anticipatory postural adjustments. It stabilizes the lumbar spine through increased intra-abdominal pressure and through continuous co-contraction with other muscles of the called *core* [15]. These adjustments are essential during sports practice because movement is continuous and continually the athletes must restore their balance with postural adjustments. However, when there is an increase in respiratory demand [18] or a decrease in the force of contraction, the postural function of the diaphragm is compromised. Kocjan et al. [26], hypothesize that diaphragmatic weakness and consequently decreased movement, create a state of insufficient irritability of proprioceptors and subsequent inadequate sensory stimuli to provide optimal postural control and maintenance of balance. Furthermore, diaphragmatic weakness can precipitate the appearance of respiratory fatigue, making it impossible for the diaphragm to perform its dual function [26].

We also have observed that in soccer players a greater number of breaths per minute in the MVV test was associated with greater length and greater anteroposterior displacement. Previous research showed that hyperventilation and consequent hypocapnia caused by high respiratory rate affects balance. Moreover, mediating vestibular compensation, hyperventilation frequently interferes with central processes and somatosensorial mechanism [27,28]. Therefore, our results confirm previous research that high respiratory rate is related to greater postural instability.

Surprisingly, our players did not show differences in static balance compared to the controls. Previous studies report that soccer players present better balance than non-athletes and even when compared to other sports disciplines. Perhaps this finding is related to the level of competition of our players, who belonged to an under-23 group, and not to a first division team. Jadczak et al. [29] showed that the more highly trained teams have better balance.

### 4.1. Implications for Practice

The results of our study can help players and coaches to improve technical skills through improved postural stability. Usually, training in this sporting discipline (as in other disciplines) does not include specific work on the inspiratory muscles. Various researchers report that general physical training has little impact on the inspiratory muscles (except swimmers) and therefore, that most athletes have these muscles untrained. The inclusion of specific inspiratory muscle training can increase the strength and endurance of these muscles and improve postural stability. Furthermore, these programs are very accessible, since they require little time and resources.

### 4.2. Limitations and Future Research

Limitations of this study include a small sample size, a single soccer team and only male players in the sample. Larger samples and the inclusion of different soccer teams, from the same professional category, could provide stronger results on the relationship between respiratory muscle function and postural stability. However, analyzing different soccer teams, apart from logistical challenges, could introduce biases due to the variability in training and physical-sporting condition of each team. Future research should consider female players and compare this relationship (respiratory muscles and postural stability) between teams of different levels, such as professionals and amateurs. In addition, it would also include the measurement of the strength of the expiratory muscles and pelvic floor, to assess the degree to which each group can contribute to postural stability.

## 5. Conclusions

Respiratory muscle function correlates with postural stability measured through center of pressure displacements. The greater the inspiratory muscle strength, the smaller the area and the lower the anteroposterior range in the closed eyes condition. On the contrary, the greater the number of breaths per minute in the voluntary maximum ventilation test, the greater the area and the greater the lateral range in the open eyes condition. Future studies with larger samples should be carried out to corroborate these results.

## Figures and Tables

**Table 1:** General characteristics of the participants.

Variables	Soccer (*n* = 18)	Control (*n* = 10)	*p*
Age (years)	20.28 ± 0.89	22.70 ± 4.29	0.11
Weight (Kg)	73.90 ± 6.49	75.32 ± 8.38	0.62
Height (m)	178.16 ± 4.90	177.10 ± 6.93	0.63
MIP (cmH_2_O)	163.88 ± 33.20	138.40 ± 28.87	0.05
MIP (%pred)	105.69 ± 22.20	90.33 ± 19.23	0.07
MVV (liters)	194.49 ± 23.44	184.64 ± 23.23	0.29
MVV (bpm)	99.37 ± 33.45	175.74 ± 26.38	<0.001
OE_length (mm)	3803.09 ± 1608.33	3308.13 ± 434.95	0.35
OE_area (mm^2^)	138.61 ± 106.38	337.63 ± 484.51	0.23
OE_X range (mm)	12.59 ± 6.03	14.78 ± 11.53	0.51
OE_Y range (mm)	14.21 ± 5.93	14.97 ± 7.04	0.76
CE_length (mm)	4028.74 ± 1552.15	3836.40 ± 642.72	0.71
CE_area (mm^2^)	111.54 ± 105.58	78.59 ± 67.65	0.38
CE_X range (mm)	11.58 ± 4.70	11.85 ± 5.29	0.89
CE_Y range (mm)	14.02 ± 6.87	14.52 ± 6.86	0.85

The data are expressed as mean ± SD; SD = standard deviation; MIP = maximal inspiratory pressure; % pred = % predicted; MVV = voluntary maximum ventilation; l = liters; bpm = breaths per minute; X range = medio-lateral range; Y range = anterior-posterior range; OE = open eyes; CE = closed eyes.

**Table 2:** Pearson’s correlation between respiratory muscle strength and stabilometric variables in soccer player and control groups.

Variables	Trials	MIP (cmH_2_0)		MIP (% pred)		MVV (l)		MVV (bpm)	
		Soccer	Control	Soccer	Control	Soccer	Control	Soccer	Control
Area (mm^2^)	OE	−0.090	−0.175	−0.110	−0.055	0.224	−0.255	0.372	0.279
	CE	−0.106	−0.108	−0.093	−0.035	0.118	0.013	0.422	0.343
Length (mm)	OE	−0.215	0.424	−0.190	0.428	0.146	−0.150	0.606 **	−0.538
	CE	−0.535 *	−0.040	−0.585 *	−0.167	0.318	−0.180	0.465	−0.570
X range (mm)	OE	0.233	0.424	0.255	0.405	0.125	−0.093	0.127	−0.423
	CE	−0.527 *	0.200	−0.560 *	0.073	0.403	−0.261	0.295	−0.306
Y range (mm)	OE	−0.290	0.155	−0.304	0.137	0.231	−0.507	0.558 *	0.000
	CE	−0.223	−0.110	−0.308	−0.245	0.259	−0.207	0.196	−0.516

X range = medio-lateral range; Y range = anterior-posterior range; OE = open eyes; CE = closed eyes; MIP = maximal inspiratory pressure; % pred = % predicted; MVV = voluntary maximum ventilation; l = liters; bpm = breaths per minute; *: The correlation is significant (*p* < 0.05); **: The correlation is significant (*p* < 0.01).

## Data Availability

All data are available under request to the corresponding author.
